# Water Quality Indicator Interval Prediction in Wastewater Treatment Process Based on the Improved BES-LSSVM Algorithm

**DOI:** 10.3390/s22020422

**Published:** 2022-01-06

**Authors:** Meng Zhou, Yinyue Zhang, Jing Wang, Yuntao Shi, Vicenç Puig

**Affiliations:** 1 School of Electrical and Control Engineering, North China University of Technology, Beijing 100144, China; zhoumeng@ncut.edu.cn (M.Z.); zyy17718301596@163.com (Y.Z.); jwang@ncut.edu.cn (J.W.); 2 Advanced Control Systems Research Group at Institutde Robòtical, CSIC-UPC, Universitat Politècnica de Catalunya-BarcelonaTech, 08028 Barcelona, Spain; vicenc.puig@upc.edu

**Keywords:** water quality monitoring, data pre-processing, improved IBES-LSSVM algorithm, interval prediction method

## Abstract

This paper proposes a novel interval prediction method for effluent water quality indicators (including biochemical oxygen demand (BOD) and ammonia nitrogen (NH3-N)), which are key performance indices in the water quality monitoring and control of a wastewater treatment plant. Firstly, the effluent data regarding BOD/NH3-N and their necessary auxiliary variables are collected. After some basic data pre-processing techniques, the key indicators with high correlation degrees of BOD and NH3-N are analyzed and selected based on a gray correlation analysis algorithm. Next, an improved IBES-LSSVM algorithm is designed to predict the BOD/NH3-N effluent data of a wastewater treatment plant. This algorithm relies on an improved bald eagle search (IBES) optimization algorithm that is used to find the optimal parameters of least squares support vector machine (LSSVM). Then, an interval estimation method is used to analyze the uncertainty of the optimized LSSVM model. Finally, the experimental results demonstrate that the proposed approach can obtain high prediction accuracy, with reduced computational time and an easy calculation process, in predicting effluent water quality parameters compared with other existing algorithms.

## 1. Introduction

Nowadays, freshwater is considered one of the most critical resources for humans, since it can ensure the availability of an acceptable quantity of water for livelihoods, health, ecosystems and production. Hence, freshwater plays a key role in poverty and disease burden reduction, economic growth and environmental sustainability [1,2]. This fact has long been acknowledged all over the world. However, due to industrial pollution, rapid population growth and farmland sewage caused by the extensive use of chemical fertilizers, pesticides and herbicides, the shortage of freshwater sources is a serious and challenging issue [3,4].

Wastewater treatment is one key technology to potentially provide additional water supplies, and it is very important for the functioning of the economy and society. Wastewater treatment has been attracting a lot of attention, since it can not only remove organic wastes to reduce the environmental burden, but also offer the advantage of producing a renewable source of water [5,6]. Wastewater treatment is a very complex process with a variety of physical and biochemical reactions since it presents nonlinear dynamic behavior, time delay and uncertainty [7]. In wastewater treatment plant processes, effluent water quality monitoring is an important task that involves measuring the evolution of the quality parameters in time.

Note that most traditional methods of measuring these quality indicators for wastewater treatment processes are based on manual lab-based monitoring approaches, with manual sample collection, long-time transportation and biological/microbial testing in a laboratory, which is cumbersome and time-consuming. Usually, the testing equipment is very expensive and cannot be used online. In addition, since the process of wastewater treatment is complex, some control strategies are necessary and required to be deployed to guarantee that effluent quality indicators behave normally. In recent decades, water quality monitoring has been evolving to the latest wireless sensor networks [8], such that most of the important indicators of effluent water (pressure, pH, level and so on) can be measured by their corresponding sensors online. However, there are still some parameters that cannot be measured quickly due to high costs and the limitations of sensors, such as BOD and NH3-N. Usually, the concentration of the BOD/NH3-N effluent associated with a wastewater treatment process is an important factor to measure the water quality since the discharge of a large amount of NH3-N and BOD wastewater will lead to water eutrophication, which can affect human health. In China’s “Pollutant Discharge Standard for Urban Wastewater Treatment Plants (GB18918-2002)”, the Class A standard stipulates that the maximum discharge for NH3-N is 5 mg/L, while for BOD, it is 10 mg/L. Thus, measuring these effluent quality indicators with high accuracy is an important issue.

Researchers have focused on soft-sensing methods to predict these effluent quality indicators and the prediction task is addressed combining data analytics and water quality control. Soft-sensing methods aim to find some certain relationships between easy-to-measure variables and difficult-to-measure variables in the sewage treatment process. Then, a suitable model is established based on these relationships, and difficult-to-measure variables can be predicted based on the soft-sensing models.

Machine learning approaches are usually considered a subset of artificial intelligence. They focus on some statistical models and algorithms to extract patterns from data so that useful inferences can be used to predict new data. Recently, with the development of machine learning, artificial neural network (ANN), support vector machine (SVM), decision tree, random forest, ensemble learning and many other methods have been researched in depth and have a wide range of applications, including text processing, computer vision, healthcare, finance and robotics. They can also be used for socio-economic and environmental studies [9,10,11,12]. In [12], the impacts of flood protection in Bangladesh were evaluated by machine learning methods. In [13], a gray model and ANN method were investigated to predict suspended matter and chemical oxygen demand in the wastewater treatment process. Cong et al. proposed a mixed soft sensor model based on a wavelet neural network and adaptive weighted fusion for the online prediction of effluent COD [14]. M. Hamada carried out the assessment of a wastewater treatment plant’s performance based on ANN and a multiple linear regression method [15]. M. Zeinolabedini et al. proved that applying various parent wavelet functions to the neural network structure can improve the accuracy of predicting the wastewater sludge volume [16]. A. K. Kadam et al. used ANN and multiple linear regression to model and predict water quality parameters in river basins [17]. S. Heddam et al. investigated a generalized regression neural network model to predict the BOD of effluent in wastewater treatment plants [18]. Tan et al. predicted the first weighting from the working face roof in a coal mine based on a GA-BP neural network [19]. V. Nourani et al. proved that the prediction ability of a neural network ensemble is more reliable [20].

Compared with the ANN method, SVM is another important prediction technique, which can effectively solve the problem of high-dimensional data model construction under the condition of limited samples, and has strong generalization ability. Hence, many scholars have carried out a lot of research on SVM-based prediction. Cheng et al. proposed a variety of kernel single-class SVMs to monitor and predict the intake conditions of wastewater treatment plants [21]. Han et al. developed a neural network model for predicting the sludge volume index based on information transfer strength and adaptive second-order algorithms [22]. Wu et al. proposed an adaptive multi-output soft sensor model for monitoring wastewater treatment and made several simulation comparisons to prove the superiority of the algorithm [23]. K. Lotfi et al. used a linear–nonlinear hybrid method to predict the effluent index of a wastewater treatment plant, which improves the prediction ability of the single method [24]. Han et al. proposed a data-based predictive control strategy and proved its superiority through several simulations [25]. In [26], the total solid content of a wastewater treatment plant was predicted by an SVM model, which can enhance performance and durability.

Although SVM is a small-sample learning method and has been widely used to solve the wastewater prediction problem, the calculation process is multifarious, which is difficult to implement for large-scale training samples [27]. To overcome these disadvantages, the least-squares support vector machine (LSSVM) has been proposed. LSSVM improves the performance of the SVM algorithm by solving linear programming rather than quadratic programming. In this way, the calculation process can be reduced and the computation speed greatly improved [28]. Zhang et al. proposed an improved LSSVM model based on SVM to predict river flow [29]. Fei Luo et al. integrated the Gustafson-Kessel algorithm and least-squares support vector machine for line prediction of [30]. D. S. Manu et al. combined SVM and an adaptive neuro-fuzzy reasoning system model to predict the effluent nitrogen content of wastewater treatment plants [31]. Liu et al. investigated the online prediction of effluent COD in an anaerobic wastewater treatment system based on principal component analysis and the LSSVM algorithm [32].

Note that there are some unknown parameters in the kernel functions of LSSVM that need to be selected in advance. Generally, these parameters are determined according to experience, which may be time-consuming, and it is difficult to find the optimal parameters. Nowadays, swarm intelligence optimization algorithms are researched extensively, since the optimal solution can be found by swarm intelligence to perform a collaborative search mechanism. The results of the combination of swarm intelligence optimization algorithms and machine learning methods can be found in a large number of references. In [33], a hybrid model of particle swarm optimization (PSO) and support vector machine is proposed to predict the turbidity and pH value of sand filtered water in irrigation systems. Han et al. use an adaptive PSO algorithm to design self-organizing radial basis function neural networks to improve the accuracy and save time [34]. Chen et al. study the artificial bee colony optimization back-propagation network to predict the water quality of a water diversion project [35]. Fan et al. use the LSSVM model to improve the performance of predicting the safety factor of a circular slope [36]. Mahdi Shariati et al. use the gray wolf algorithm to optimize ELM model parameters to predict the compressive strength of partially replaced cement concrete [37]. However, to the best of the authors’ knowledge, these swarm intelligence methods may fall into local optima and do not find the global optimal solutions.

Most of the above-mentioned methods only focus on point prediction, without providing information regarding accuracy. The prediction results have strong uncertainty that affects the decision-making process, increasing the risk of not making good decisions. Prediction interval (PI) is a standard tool for quantifying prediction uncertainty. PI not only provides the range where the target value is most likely to exist, but also indicates its accuracy. Yao et al. combined the mean variance estimation (MVE) method with a recurrent neural network to measure the uncertainty in prediction [38]. Yuan et al. combined beta distribution with the PSO-LSTM model to obtain the wind power prediction interval with high reliability and a narrow interval width, so as to provide decision support for the safe and stable operation of power systems [39]. Liao et al. combined the bootstrap method with the long and short memory network to realize the uncertain prediction of the remaining service life of the machine [40]. Marin et al. obtained the prediction interval of power consumption by combining the delta method with a fuzzy prediction model [41]. Sun et al. constructed a high-quality prediction interval based on the two-step method of dual ELM and applied it to the scheduling of a gas system [42]. In recent years, a direct interval prediction method called upper and lower bound estimation (LUBE) has been proposed. The main idea of this method is to directly construct the upper and lower bounds of PI by optimizing the coefficients of the neural network according to the interval quality evaluation index. This approach can provide good performance and does not consider strict data distribution assumptions, such that it can provide more information about the prediction results, which motivates the work of this paper.

The main objective of this paper is to obtain a soft-sensor-based interval prediction method with high prediction accuracy and less computational time to predict the effluent water quality parameters, which is significant for water quality monitoring and control. Aiming at the online prediction of BOD/NH3-N effluent in a wastewater treatment plant within a smart data-driven framework, the main contributions of this paper are the following:

- Data pre-processing methods, i.e., abnormal data elimination and normalization, are taken into consideration after the data and their related auxiliary variables are collected. Then, some key factors of the wasterwater quality indicators are selected based on the gray correlation analysis algorithm.
- In order to improve the prediction accuracy of BOD/NH3-N effluent, a novel IBES-LSSVM algorithm is proposed, in which an improved bald eagle search (IBES) optimization algorithm is used to find the optimal parameters of the least-squares support vector machine (LSSVM). The superiority of the proposed method is verified by comparing it with the existing soft-sensing models (such as GWO, WOA, PSO, SSA) using some benchmark functions and providing higher prediction accuracy.
- In order to estimate the uncertainty of the model prediction results and make better decisions, after obtaining the point prediction results, the interval prediction bounds of effluent quality are also generated. Compared with some existing soft-sensing models, the proposed interval prediction method can obtain a more accurate prediction range.

The structure of this paper is as follows: In Section 2, the problem description is given, including the real data collection, data pre-processing and gray-correlation-analysis-based data selection. Section 3 describes the model uncertainty analysis by using the proposed IBES-LSSVM algorithm and LUBE algorithm. In Section 4, the simulation examples are depicted, demonstrating the effectiveness of the proposed method based on the BOD and NH3-N data. Section 5 draws the main conclusions of this paper.

## 2. Problem Description

**Figure 1:**
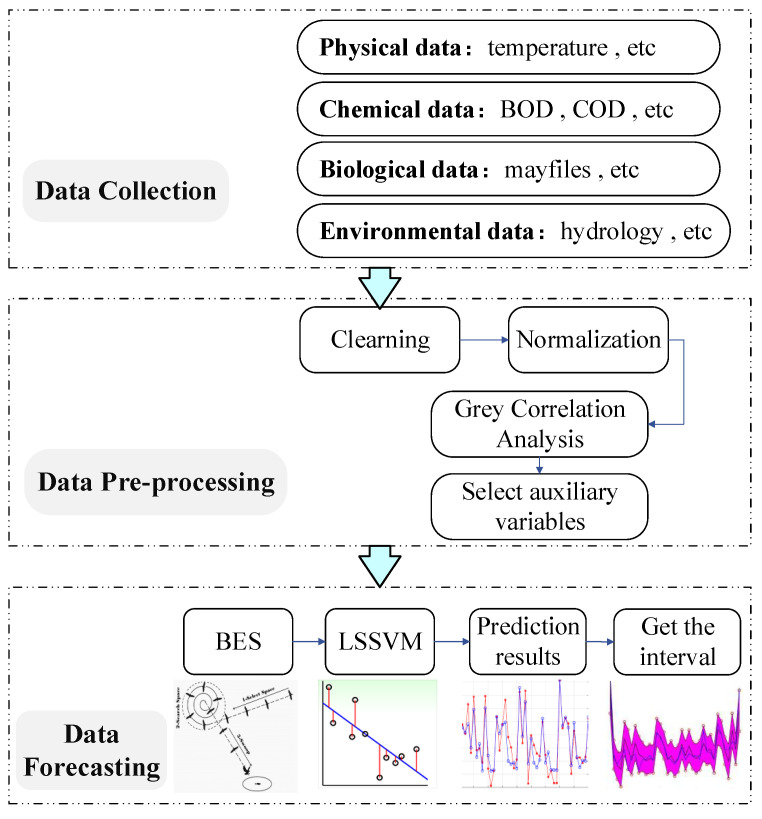
Main steps of the proposed approach.

In this paper, a soft-sensing-based method is investigated to analyze and predict the water quality indicators, including three main aspects: data collection, data pre-processing and data interval prediction. The main steps of the approach presented in this paper are shown in Figure 1.

Under a smart data-driven framework, in order to predict water quality tendencies and analyze the mechanisms behind the considered data sources, enough relevant experimental data in real time must be collected based on the prediction quality indicators. Most collected data may present several issues, such as data sparsity and data synchronization, among others. After the data are collected, they must be pre-processed in advance by applying several procedures, such as data cleaning, abnormal data elimination or normalization. Then, correlation analysis from different dimensions of water quality indicators should be considered to extract the relations between these auxiliary variables and find the key factors.

### 2.1. Data Collection

Due to the complexity of the wastewater treatment process and the large number of parameters that need to be set, it is necessary to determine the characteristic variables related to the water quality to be determined as auxiliary variables. The data that can evaluate the quality or impact of water quality in wastewater treatment plants are mainly divided into the following four categories [43]:

- **Physical data**: Physical properties are the ones that must be monitored throughout the treatment process, including total suspended solids, temperature, conductivity, transparency, total dissolved solids, etc.
- **Chemical data**: Chemical water quality indices of the national comprehensive discharge standard for water pollutants, including: pH, biochemical oxygen demand, biochemical oxygen consumption, heavy metals, nitrates, etc.
- **Biological data**: Biomarkers include a variety of microorganisms in the water, such as mayflies, E. coli, etc.
- **Environmental data**: Environmental data cover the whole process of water supply, including indexes of weather, hydrology, soil or ecology.

This paper focuses on a real wastewater treatment plant in Beijing, China, from August 2014 to September 2014 [7,44]. Two data sets are collected first, which are used to predict the BOD/NH3-N effluent, separately. (1) BOD data set: containing 360 batches of data with 23 variables (including the BOD effluent parameters)—the detailed information is shown in Table 1; (2) NH3-N data set: including 10 characteristic variables related to NH3-N effluent parameters, as shown in Table 2.

### 2.2. Elimination of Abnormal Data

Data collected from wastewater treatment plants can contain erroneous values because of improper instrument operation, human or environmental interference and other factors. As a result, we need to analyze the collected data first, and eliminate some abnormal or meaningless data.

In this paper, we use the 3σ criterion to handle the abnormal data of the two collected data sets. The sample data are denoted as $x_{1},x_{2},\cdots ,x_{n}$. $\eta_{i}$ is used to represent the data residual error. Then, the standard deviation is calculated as follows:

(1) $$\sigma =\sqrt{\frac{\sum_{i=1}^{n}{\eta_{i}}^{2}}{n-1}}$$

(2) $$\eta_{i}=x_{i}-\bar{x}$$

where *n* represents the number of elements in the data set, and $\bar{x}$ is the data average.

If the residual error of particular data sample $x_{i}$ satisfies

(3) $$\left|\eta_{i}\right|>3\sigma$$

this means that it corresponds to an abnormal sample and needs to be eliminated. Otherwise, $x_{i}$ is accepted.

**Table 1:** Effluent BOD data set.

Number	Auxiliary Variable	
01	Influent pH (IPH)	
02	Effluent pH (EPH)	
03	Influent SS	(mg/L)
04	Effluent SS (ESS)	(mg/L)
05	Influent BOD (IBOD)	(mg/L)
06	Influent COD (ICOD)	(mg/L)
07	Effluent COD (ECOD)	(mg/L)
08	Sludge settling ratio of biochemical tank	(mg/L)
09	MLSS in biochemical tank (MLSS)	(mg/L)
10	Biochemical pool Do	(mg/L)
11	Influent oil (IOil)	(mg/L)
12	Effluent oil (EOil)	(mg/L)
13	Influent NH3-N (INH3-N)	(mg/L)
14	Effluent NH3-N	(mg/L)
15	Influent Chroma (IC)	(d)
16	Effluent Chroma (EC)	(d)
17	Influent TN (IT)	(mg/L)
18	Effluent TN	(mg/L)
19	Influent phosphate concentration (IPC)	(mg/L)
20	Effluent phosphate concentration	(mg/L)
21	Inlet water temperature	(${}^{\circ }$C)
22	Outlet water temperature	(${}^{\circ }$C)
23	Effluent BOD (EBOD)	(mg/L)

**Table 2:** Effluent NH3-N data set.

Number	Auxiliary Variable	
01	Effluent TP	(mg/L)
02	Influent TP (ITP)	(mg/L)
03	Temperature (T)	(${}^{\circ }$C)
04	Anaerobic terminal ORP (ATORP)	(mv)
05	Aerobic front end DO	(mg/L)
06	Aerobic terminal DO	(mg/L)
07	Total suspended solids TTS (TTS)	(mg/L)
08	Effluent PH (EPH)	
09	Effluent ORP (EORP)	(mL)
10	Effluent nitrate (EN)	(mg/L)
11	Effluent NH3-N (ENH3-N)	(mg/L)

### 2.3. Data Normalization

Different variables often have different dimensions and dimensional units. In order to eliminate the dimensional influence between indicators, it is necessary to normalize the data to achieve uniformity among the different data indicators. There are four classes of normalization methods, i.e., rescaling, mean normalization, standardization and scaling to unit length. In this paper, the rescaling method is selected. The normalization formula is as follows:

(4) $${\tilde{x}}_{i}=\frac{x_{i}-x_{i\min}}{x_{i\max}-x_{i\min}}$$

where $x_{i}$ is any value of a variable; $x_{i\min}$ and $x_{i\max}$ are, respectively, the minimum and maximum value of the variable.

After this kind of normalization, all the values of the data are set in the range of $\left[0,1\right]$.

### 2.4. Correlation Degree Analysis

Since different characteristic variables will have different influences on the predicted variables, to obtain a soft-sensing model with a simpler structure, it is necessary to choose the quality indicators with high correlations. Selecting $\overset{´}{m}$ auxiliary variables from *m* variable, it has $\overset{´}{m}<m$. In practice, the larger *m* is, the smaller $\overset{´}{m}$ is compared to *m*.

In this paper, the gray relational degree analysis method is investigated to select the characteristic variables of BOD and NH3-N effluents. Gray relational degree analysis is a multi-factor statistical method, which describes the strength of the relationship between various factors according to the gray relational degree. This method looks for the inconsistency between quantitative results and quantitative analysis in the traditional mathematical statistics method and reduces the amount of calculation.

The gray correlation coefficient is formulated as follows:

(5) $$\beta =\left|x_{0}\left(k\right)-x_{j}\left(k\right)\right|$$

(6) $$\mu_{j}\left(k\right)=\frac{\min_{j}\min_{k}\beta +\rho \cdot \max_{j}\max_{k}\beta }{\beta +\rho \cdot \max_{j}\max_{k}\beta }$$

where *j* means the *j*-th variable, *k* is the *k*-th iteration, $x_{0}\left(k\right)$ is the output variable, $x_{j}\left(k\right)$ is the input variable, $\mu_{j}$ is the gray correlation coefficient and ρ is the resolution coefficient. If ρ is smaller, the difference between correlation coefficients is larger, and the distinguishing ability is stronger.

Then, the gray correlation degree can be calculated as follows:

(7) $$\gamma_{j}=\frac{1}{n}\sum_{k=1}^{n}\mu_{j}\left(k\right)$$

where *n* is the number of variables.

If the gray correlation degree is larger, this means that the corresponding variable has a higher correlation with the effluent quality indicators. Then, according to the gray correlation degree, the characteristic variables are sorted from front to back. Usually, a threshold is determined in advance as *ħ*, and then the key indicators can be selected as the input of the soft-sensing model if

(8) $$\gamma_{j}>\hbar$$

is satisfied.

## 3. Methodology

**Figure 2:**
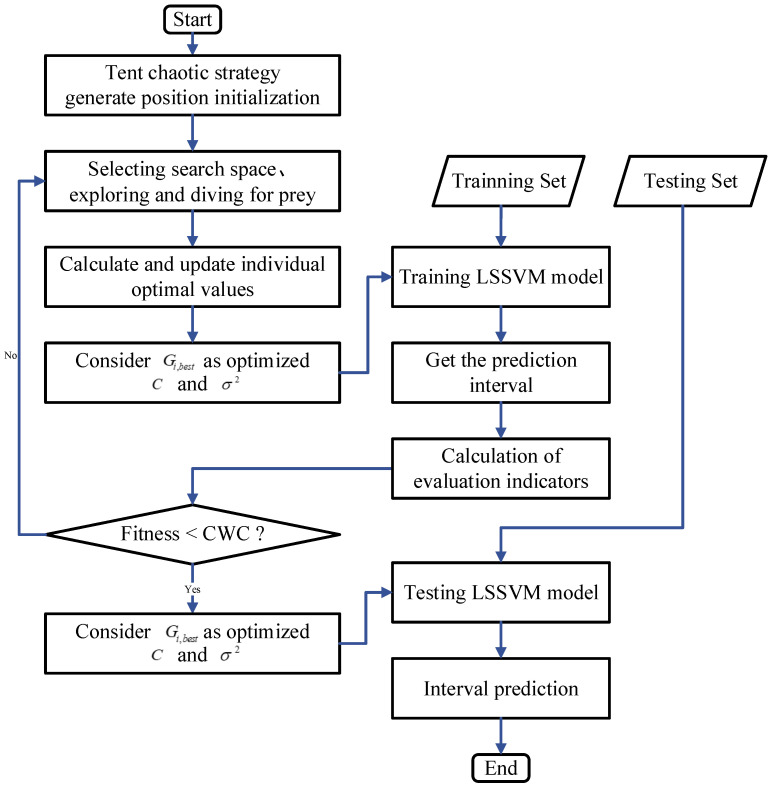
Flow chart of IBES-LSSVM model.

In this section, a novel IBES-LSSVM method is proposed to find the optimal kernel function parameters of the LSSVM in Figure 2.

### 3.1. LSSVM Algorithm

The theory of LSSVM was first proposed by Suykens in 1994. LSSVM is a kernel learning machine following the principle of structural risk minimization and is suitable for analyzing the issue of sample classification and regression estimation [45].

In LSSVM theory, firstly, the sample data are mapped to higher dimensions through nonlinear changes, and linear functions are used for fitting in this high-dimensional feature space:

(9) y(x)=w·ϕ(x)+b

where y(x) is the output variable, *x* is the input variables, and *w* and *b* are weight and bias terms, respectively.

The optimization objectives of the LSSVM regression algorithm can be formulated as

(10) $$\begin{array}{c} \min J(w,\xi_{i})=\frac{1}{2}w^{T}w+\frac{C}{2}\sum_{i=1}^{n}\xi_{i}^{2}\\ \;s.t.\\ \;\;y_{i}=w\cdot \phi (x)+b+\xi_{i},\;\;\;\;i=1,2,\cdots ,n \end{array}$$

where *C* is the regularization coefficient, $\xi_{i}$ is the relaxation variable, and $\sum_{i=1}^{n}\xi_{i}^{2}$ is the experience risk.

By means of Lagrange multipliers $\alpha_{i}$, (10) can be expressed as:

(11) $$\begin{array}{cc} L(w,b,\xi_{i},\alpha_{i}) & =\frac{1}{2}w^{T}w+\frac{C}{2}\sum_{i=1}^{N}\xi_{i}^{2}\\ \; & -\sum_{i=1}^{n}\alpha_{i}[w\cdot \phi (x)+b+\xi_{i}-y_{i}] \end{array}$$

According to Karush–Kuhn–Tucker (KKT) optimization conditions:

(12) $$\left\{\begin{array}{c} \frac{\partial L}{\partial b}=0\Rightarrow \sum_{i=1}^{n}\alpha_{i}=0\\ \frac{\partial L}{\partial w}=0\Rightarrow w=\sum_{i=1}^{n}\alpha_{i}\phi (x_{i})\\ \frac{\partial L}{\partial \xi_{i}}\;=\;0\Rightarrow \alpha_{i}=C\xi_{i}\\ \frac{\partial L}{\partial a}=0\Rightarrow w\cdot \phi (x_{i})+b+\xi_{i}-y_{i} \end{array}\right.\;$$

By defining kernel functions, the optimization problem (11) can be transformed into a linear solution issue:

(13) $$\left(\begin{array}{cccc} 0 & 1 & \cdots & 1\\ 1 & \frac{K(x_{1},x_{1})+1}{C} & \cdots & K(x_{1},x_{n})\\ \vdots & \vdots & & \vdots \\ 1 & K(x_{n},x_{1}) & \cdots & \frac{K(x_{n},x_{n})+1}{C} \end{array}\right)\left(\begin{array}{c} b\\ \alpha_{1}\\ \vdots \\ \alpha_{n} \end{array}\right)=\left(\begin{array}{c} 0\\ y_{1}\\ \vdots \\ y_{n} \end{array}\right)$$

where $K(x,x_{i})$ is the kernel function.

The Lagrange multiplier and its parameters can be obtained from (13). Therefore, the output of LSSVM can be obtained:

(14) $$\hat{y}\left(x\right)=\sum_{i=1}^{n}\alpha_{i}K\left(x,x_{i}\right)+b\;$$

For LSSVM, there are many different types of kernel functions, such as linear function, polynomial kernel function, radial basis function (RBF), sigmoid kernel function, etc. Different kernel functions will produce difference types of LSSVM. In this paper, we select RBF as the kernel function of the model:

(15) $$K(x,x_{i})=\exp\left(-\frac{{∥x-x_{i}∥}^{2}}{2\sigma^{2}}\right)$$

where σ is the variance of RBF.

Through the aforementioned analysis, LSSVM has two tunable parameters (regularization coefficient *C* and variance of radial basis kernel function σ with RBF), which are important and need to be determined. To obtain the optimal two parameters, the next step is to use an improved PSO algorithm to optimize them.

### 3.2. IBES-LSSVM Algorithm

The BES algorithm is an optimization algorithm that simulates the hunting strategy of vultures when looking for fish. It can obtain a single optimal solution through multiple iterations and finally obtain the overall optimal solution, such that the position of the optimal solution corresponds to the optimal parameter value.

BES hunting is divided into three stages. In the first stage (selection space), the eagle selects the space with the largest prey number. In the second stage (spatial search), the eagle moves in the selected space to find the prey. In the third stage (dive), the eagle swings from the best position determined in the second stage and determines the best hunting.

In the selection stage, firstly, this paper optimizes the initial prey position and adopts the tent chaos strategy, which has the advantages of simple structure and strong ergodicity. Then, the linear decreasing method is used to improve the control parameters of the vulture iterative update position. The optimal model parameters of the model can be found that improve the quality of the fitting. The tent chaotic mapping function is described as:

(16) $$P_{i+1}=\left\{\begin{array}{cc} P_{i}/\lambda , & P_{i}\in \left[0,\lambda \right)\\ \left(1-P_{i}\right)/\left(1-\lambda \right), & P_{i}\in \left[\lambda ,1\right] \end{array}\right.$$

where λ is [0,1].

Then, the vultures hunt for food. The formula is:

(17) $$P_{\mathrm{new},i}=P_{\mathrm{best}}+R_{1}\cdot C_{1}\cdot \left(P_{\mathrm{mean}}-P_{i}\right)$$

where $R_{1}$ is a parameter controlling the position change, and $C_{1}$ is a random number between (0,1). $P_{best}$ is the current optimal location. $P_{mean}$ is the average distribution location of vultures after the previous search. $P_{i}$ is the location of the *i*-th vulture.

In the search phase, vultures search for prey in the selected search space and move in different directions in the spiral space to speed up the search. The best position for subduction is:

(18) $$P_{i,\;\mathrm{new}\;}=P_{i}+b\left(i\right)\cdot \left(P_{i}-P_{i+1}\right)+a\left(i\right)\cdot \left(P_{i}-P_{\mathrm{mean}\;}\right)$$

where:

(19) $$a\left(i\right)=\frac{ar(i)}{\max(|ar|)}$$

(20) $$b\left(i\right)=\frac{br(i)}{\max(|br|)}$$

(21) ar(i)=r(i)·sin[(θ(i))]

(22) br(i)=r(i)·cos[(θ(i))]

(23) $$r\left(i\right)=\theta \left(i\right)+R_{2}\cdot C_{3}$$

(24) $$\theta \left(i\right)=\pi \cdot \omega \cdot C_{2}$$

(25) $$\omega ={\left(1-\frac{i}{i_{max}}\right)}^{2}\cdot \left(\omega_{max}-\omega_{min}\right)+\omega_{min}$$

where θ(i) and r(i) are the polar angle and polar diameter of the spiral equation, respectively. ω and $R_{2}$ are the parameters controlling the spiral trajectory. $C_{2}$ and $C_{3}$ are a random number within (0,1). The a(i) and b(i) represent the position of the vulture in polar coordinates, and the values are (−1,1).

During the dive phase, vultures swing from the best position in the search space to their target prey. All points also move towards the best point according to

(26) $$\begin{array}{cc} P_{i,\;\mathrm{new}\;}= & C_{4}\cdot P_{\mathrm{best}\;}+a_{1}\left(i\right)\cdot \left(P_{i}-R_{3}\cdot P_{\mathrm{man}\;}\right)\\ \; & +b_{1}\left(i\right)\cdot \left(P_{i}-R_{4}\cdot P_{\mathrm{best}\;}\right) \end{array}$$

where:

(27) $$a_{1}\left(i\right)=\frac{ar(i)}{\max(|ar|)}$$

(28) $$b_{1}\left(i\right)=\frac{br(i)}{\max(|br|)}$$

(29) ar(i)=r(i)·sinh[(θ(i))]

(30) br(i)=r(i)·cosh[(θ(i))]

(31) r(i)=θ(i)

(32) $$\theta \left(i\right)=\pi \cdot \omega \cdot C_{5}$$

where $R_{3}$ and $R_{4}$ represent the moving speed of the vulture to the optimal point. $C_{4}$ and $C_{5}$ are random numbers within (0,1).

### 3.3. Interval Prediction

The traditional point prediction cannot deal with the uncertainty in the operation of the system. In order to obtain the numerical estimation and its reliability, the practical application requires the calculation of the prediction interval. Interval prediction indicates the estimation interval of the range of predicted values in a certain confidence interval. Therefore, the prediction interval is composed of the upper and lower line of prediction, which provides its accuracy within a certain confidence level. Assuming that the confidence level is (1−μ)%, *l* and *u* are the lower and upper limits, respectively, when P(l<y<u)=1−μ%, and PI can be expressed as [l,u]. For a given confidence interval, the smaller the range of prediction interval, the smaller the uncertainty of prediction and the higher the accuracy.

The evaluation indexes of interval prediction are as follows [46].

PICP: The ratio of the real value to the upper and lower bounds of the prediction interval

(33) $$PICP=\frac{1}{n}\sum_{i=1}^{n}c_{i}$$

If the predicted value is within the $\left[l_{i},u_{i}\right]$ range, $c_{i}$ is 1. Otherwise, $c_{i}$ is 0. If all predicted values are included in the prediction interval, PICP = 100%. *n* is the number of prediction points. In theory, PICP⩾(1−μ)%; otherwise, PI is invalid or unreliable. When comparing the PIs by the model, the other indexes should be as small as possible under the condition that the PICP is as close to the confidence level as possible.

PINAW: The narrow PI has more information and practical value than the wide PI according to

(34) $$PINAW=\frac{1}{nR}\sum_{i=1}^{n}\left(u_{i}-l_{i}\right)$$

where *R* is the range of predicted values, respectively.

PINRW: Represents the standard square root width of the predicted interval. The expression is:

(35) $$PINRW=\frac{1}{R}\sqrt{\frac{1}{n}\sum_{i=1}^{n}{\left(u_{i}-l_{i}\right)}^{2}}$$

CWC: In practical application, it is often hoped that a narrow prediction interval width can still be obtained under the condition of high prediction probability, i.e., the prediction interval range probability and interval width will conflict. Therefore, the comprehensive index CWC is proposed:

(36) $$CWC=PINAW\left(1+\varrho \left(PICP\right)\cdot e^{-\tau \cdot (PICP-(1-\mu ))}\right)$$

where τ and μ are constants.

When working with training data, the set ϱ(PICP) is 1. In addition, in data verification, ϱ(PICP) is a step function:

(37) $$\varrho =\left\{\begin{array}{cc} 0 & PICP\geq 1-\mu \\ 1 & PICP<1-\mu \end{array}\right.$$

LUBE is a method based on neural networks to directly calculate the lower and upper bound of the prediction interval. Assuming that the two node values of the output layer of the neural network are the upper and lower limits of the interval, respectively, all the predicted values are included in this range at the confidence level (1−μ)%. The training purpose of a neural network is to minimize the objective function CWC. In this way, the probability and width of the prediction interval are considered at the same time, and the advantages and disadvantages of the prediction interval PI can be comprehensively evaluated.

The flow-chart of the proposed IBES-LSSVM algorithm is shown in Figure 2, which mainly includes the procedure presented in Algorithm 1.
**Algorithm 1** LUBE interval prediction based on IBES-LSSVM model.**Input**:
Measured data of wasterwater treatment plant.**Output**:
Prediction interval of BOD/NH3-N effluent.Step 1:Abnormal data elimination, normalization of the data according to Equations (1)–(4).Step 2:Analyzing and selecting the key indicators with high correlation degree by Equations (5)–(8).Step 3:The bald eagle population is initialized by tent chaos strategy based on Equation (16).Step 4:Local optimal solution.1:for all $X_{i}$ do:2:   for all $X_{i}$ do:3:      Obtain predicted value by means of Equations (9)–(15), (17).4:   end for5:   Using confidence, mean, standard deviation and other parameters, the prediction interval is obtained according to norminv() formula.6:   Evaluate interval fitness by means of Equations (33)–(37).7:end for8:Obtain the local optimal solution.Step 5:Global optimal solution.1:While *t* ≤ iter do:2:   for all $X_{i}$ do:3:      Update parameter *X*, *C*, σ by using Equations (18)–(25).4:      Obtain different predictions by using Equations (9)–(15).5:   end for6:   Using confidence, mean, standard deviation and other parameters, the prediction interval is obtained according to norminv() formula.7:   Judge and update by Equations (33)–(37).8:   for all $X_{i}$ do:9:      Update parameter *X*, *C*, σ by using Equations (26)–(32).10:      Obtain different predictions by using Equations (9)–(15).11:      Using confidence, mean, standard deviation and other parameters, the prediction interval is obtained according to norminv() formula.12:      Judge and update by means of Equations (33)–(37).13:   end for14:   t=t+115:end while16:Obtain the global optimal solution.Step 6:Return the global optimal prediction interval.Step 7:Output *C*, σ, fitness and other index values by using Equations (33)–(37), (38)–(41).

## 4. Simulation Results

In this section, the data sets of BOD/NH3-N effluents are collected from a wastewater treatment plant in Beijing and are used to verify the effectiveness of the proposed approach.

The following evaluation indices of several certainty point predictions are evaluated as follows:

(38) $$MSE=\frac{1}{n}\sum_{i=1}^{n}{\left({\hat{y}}_{i}-y_{i}\right)}^{2}$$

(39) $$RMSE=\sqrt{\frac{1}{n}\sum_{i=1}^{n}{\left({\hat{y}}_{i}-y_{i}\right)}^{2}}$$

(40) $$MAE=\frac{1}{n}\sum_{i=1}^{n}\left|{\hat{y}}_{i}-y_{i}\right|$$

(41) $$R^{2}=1-\frac{\sum_{i=1}^{n}{\left(\hat{y}-y_{i}\right)}^{2}}{\sum_{i=1}^{n}{\left(\hat{y}-\bar{y}\right)}^{2}}=\frac{\sum_{i=1}^{n}{\left(y_{i}-\bar{y}\right)}^{2}}{\sum_{i=1}^{n}{\left(\hat{y}-\bar{y}\right)}^{2}}$$

### 4.1. Experiment of Benchmark Functions

The proposed approach is based on the six functions listed in Table 3 with the corresponding ranges and parameters. The range is the boundary of the function search space.

**Figure 3:**
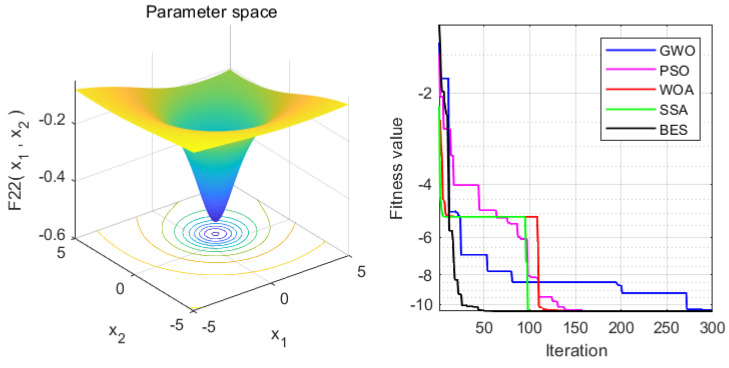
The result of F1.

**Figure 4:**
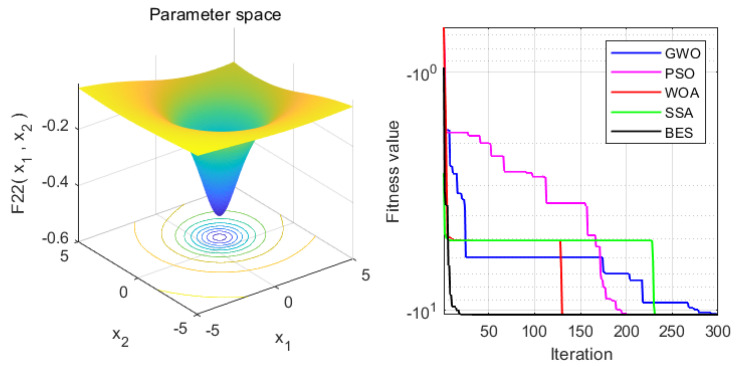
The result of F2.

**Figure 5:**
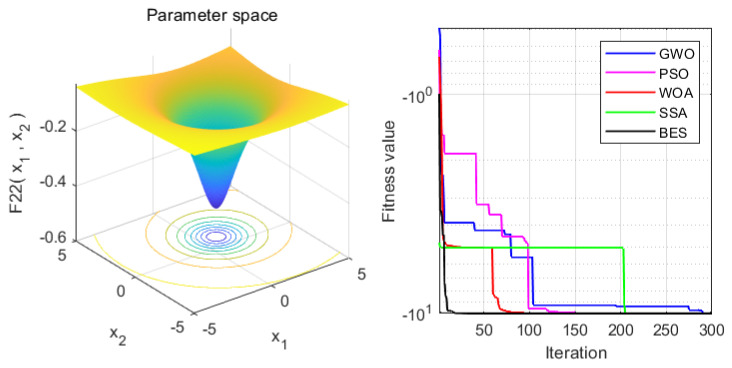
The result of F3.

**Figure 6:**
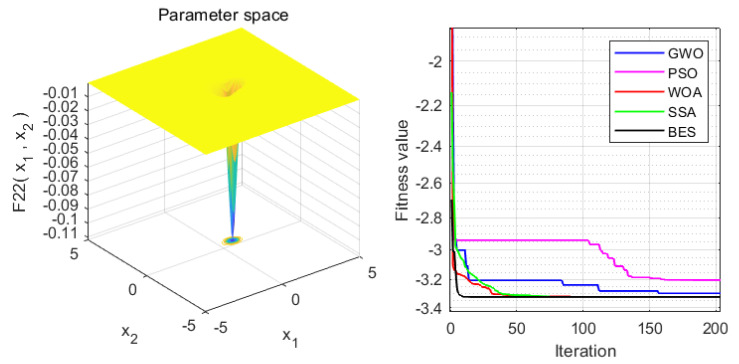
The result of F4.

**Figure 7:**
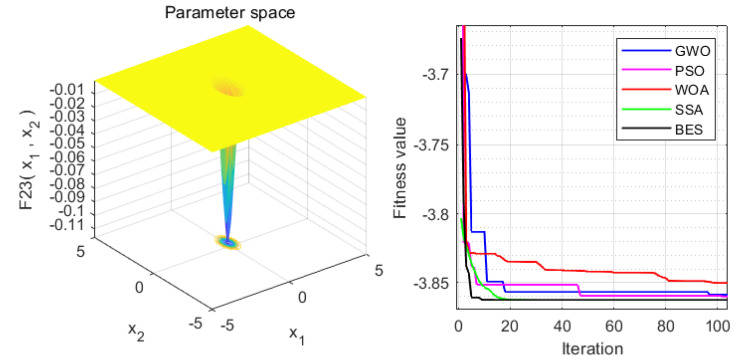
The result of F5.

**Figure 8:**
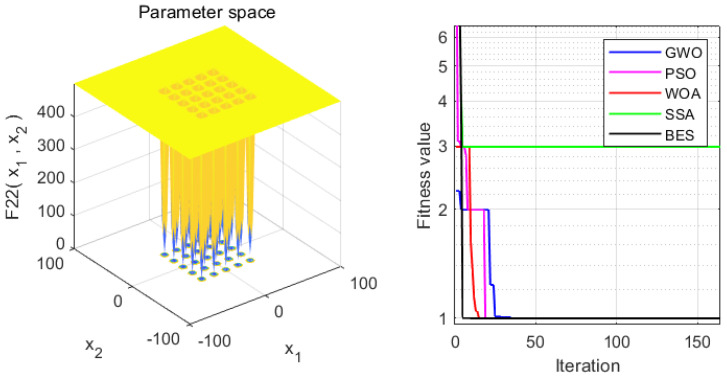
The result of F6.

In order to verify the superiority of the proposed approach, it is compared with the WOA, GWO, PSO and SSA algorithms. Statistical results are presented in Table 4. Moreover, the iteration process is depicted in Figure 3, Figure 4, Figure 5, Figure 6, Figure 7 and Figure 8. From the results, we can see that the convergence rate of IBES is better than that of the other algorithms and the proposed IBES method is able to provide competitive results on the benchmark functions.

### 4.2. Experiment of BOD Data

**Figure 9:**
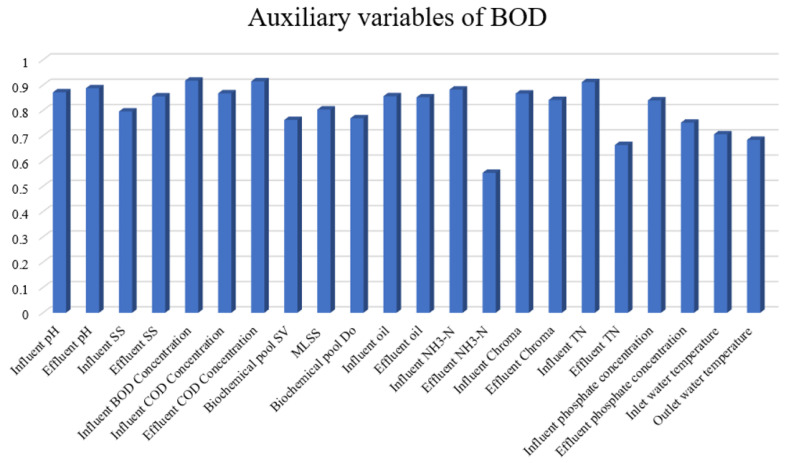
Auxiliary variables of BOD.

**Figure 10:**
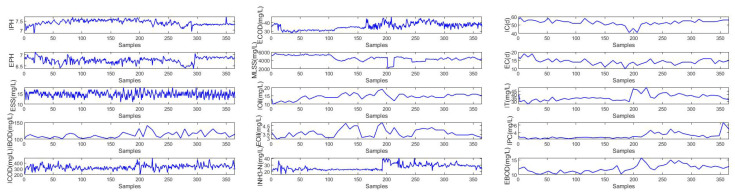
Original data of BOD.

BOD is one of the most important effluent quality indexes and can reflect the water pollution situation [7]. First, the key auxiliary variables are selected for the BOD effluent data set by calculating the gray correlation degree based on (7). The threshold of the gray correlation degree is chosen as 0.8. Hence, 14 auxiliary variables (as shown in Table 5) are selected as the soft measurement model inputs. Including the output effluent BOD, there are 15 key indicators; the detailed information is shown in Figure 9. Moreover, the description of each datum is given in Figure 10.

In this paper, the BOD effluent data set has 365 sets of data; among them, 335 sets of data are randomly selected as training samples, and the remaining 30 sets of data are treated as the prediction samples. In order to demonstrate the superiority of the proposed IBES-LSSVM method, it is compared with some existing results, i.e., CNN, LSTM, ELMAN, WOA-LSSVM, GWO-LSSVM, PSO-LSSVM and SSA-LSSVM. In the experiments, the initialization conditions are set as: iter is 50, n=30, $\omega_{max}=10$, $\omega_{min}=0$, $R_{1}=1.8$, $R_{2}=1$, $R_{3}=1.5$, $R_{4}=1.5$.

**Figure 11:**
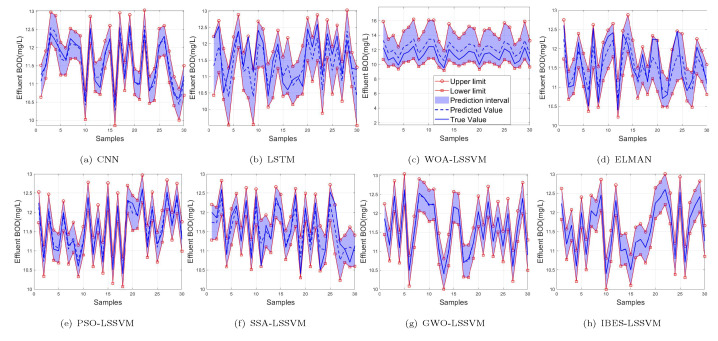
99% of BOD.

**Figure 12:**
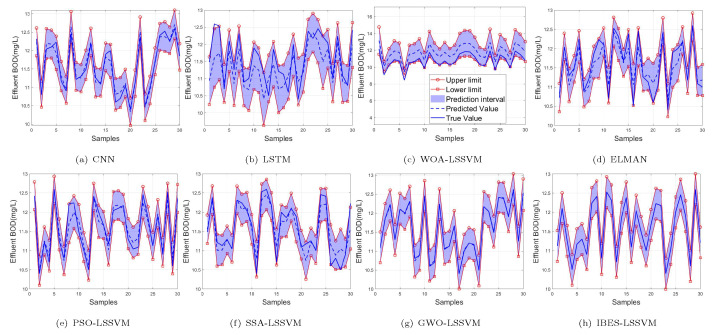
95% of BOD.

**Figure 13:**
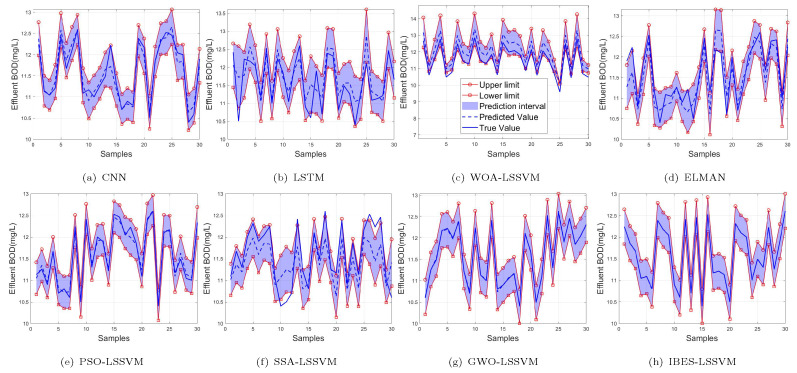
90% of BOD.

From Table 6 and Table 7 and Figure 11, Figure 12 and Figure 13, we can see that, compared with the existing CNN model, LSTM model, ELMAN model, WOA-LSSVM model, GWO-LSSVM model, PSO-LSSVM model and SSA-LSSVM model, the prediction accuracy of the proposed method is better, demonstrating its effectiveness.

### 4.3. Experiment of NH3-N Data

**Figure 14:**
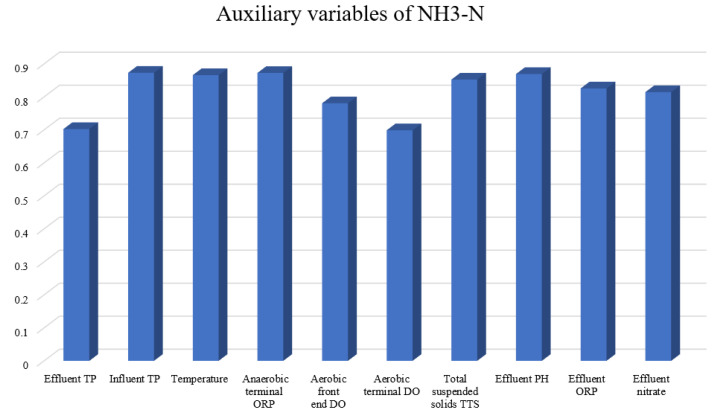
Auxiliary variables of NH3-N.

**Figure 15:**
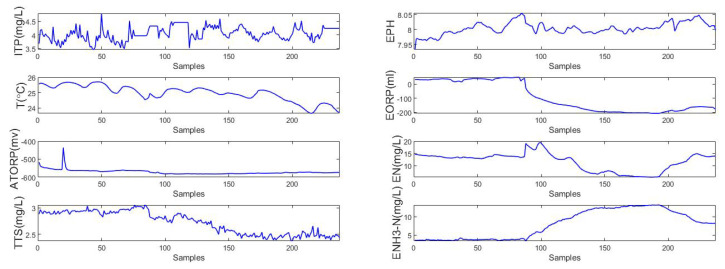
Original data of NH3-N.

In this experiment, the NH3-N effluent data set is considered, which has been described in [44]. First, the gray correlation degree is calculated from (7), and the results are presented in Figure 14. In addition, each selected auxiliary datum of the NH3-N data set is shown in Figure 15.

In this example, the threshold of the gray correlation degree is also chosen as 0.8; hence, 7 auxiliary variables (as shown in Table 8) are selected as the soft measurement model input. The experimental data of effluent NH3-N used in this paper are from a sewage treatment plant in Beijing. In total, 237 sets of data were obtained, including 200 sets of data that were randomly selected as training samples, and the remaining 37 sets of data were treated as the prediction samples.

In order to demonstrate the superiority of the proposed BES-LSSVM method, it is compared with some existing approaches, i.e., CNN, LSTM, ELMAN, WOA-LSSVM, GWO-LSSVM, PSO-LSSVM and SSA-LSSVM. In the experiments, the parameters are set as follows: iter is 50, n=30, $\omega_{max}=10$, $\omega_{min}=0$, $R_{1}=1.8$, $R_{2}=1.2$, $R_{3}=1.8$, $R_{4}=1.8$.

**Figure 16:**
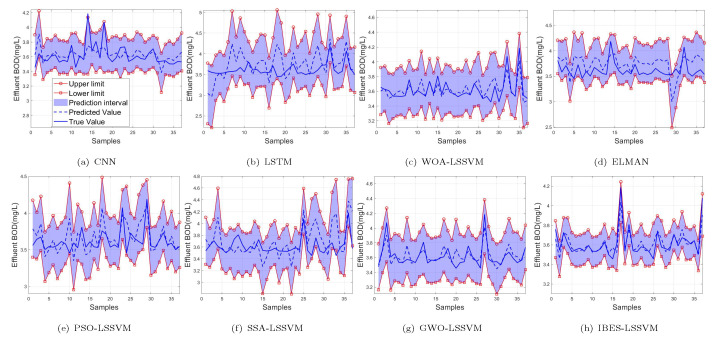
99% of NH3-N.

**Figure 17:**
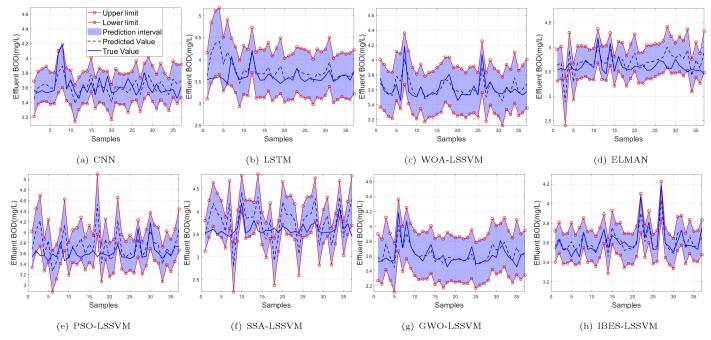
95% of NH3-N.

**Figure 18:**
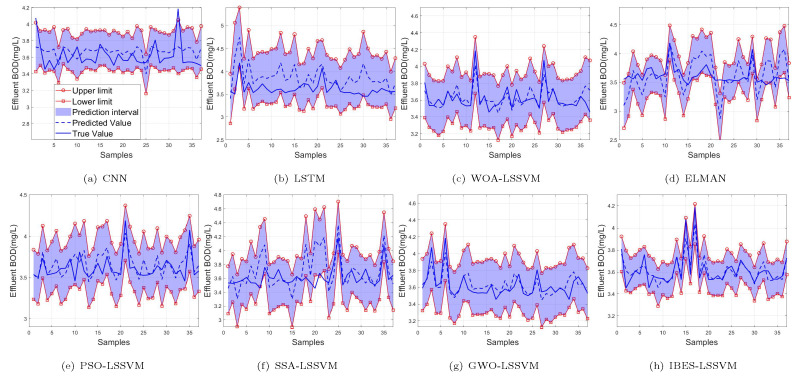
90% of NH3-N.

From Table 9 and Table 10 and Figure 16, Figure 17 and Figure 18, we can see that, compared with the existing CNN model, LSTM model, ELMAN model, WOA-LSSVM model, GWO-LSSVM model, PSO-LSSVM model and SSA-LSSVM model, the prediction accuracy of the proposed method is the best, demonstrating its effectiveness.

## 5. Conclusions

This paper investigates an improved IBES-LSSVM algorithm to predict the effluent water quality indicators of a wastewater treatment plant, in which an improved BES method is proposed to find the optimal LSSVM parameters. To deal with the uncertainties of the data, the prediction interval is generated within a certain confidence level, which could provide the upper and lower bounds of the prediction results. Compared with other existing methods, the proposed approach demonstrates high prediction accuracy, with reduced computational time and an easy calculation process, in predicting effluent water quality parameters. Note that the proposed results can only predict the water quality indicators, but this is not the end work for a wastewater treatment plant process. The application of this work to reliable decision-making and the generation of a suitable control strategy will be our future work. 

## Figures and Tables

**Table 3:** Benchmark functions.

	Function	Range	Parameters
F1	$F\left(x\right)=-\sum_{i=1}^{10}{\left[\left(X-a_{i}\right){\left(X-a_{i}\right)}^{T}+c_{i}\right]}^{-1}$	[1,10]	dim = 4 popsize = 100 iteration = 300
F2	$F\left(x\right)=-\sum_{i=1}^{7}{\left[\left(X-a_{i}\right){\left(X-a_{i}\right)}^{T}+c_{i}\right]}^{-1}$	[1,10]	dim = 4 popsize = 100 iteration = 300
F3	$F\left(x\right)=-\sum_{i=1}^{5}{\left[\left(X-a_{i}\right){\left(X-a_{i}\right)}^{T}+c_{i}\right]}^{-1}$	[1,10]	dim = 4 popsize = 100 iteration = 300
F4	$F\left(x\right)=-\sum_{i=1}^{4}c_{i}\exp\left(-\sum_{j=1}^{6}a_{ij}{\left(x_{j}-p_{ij}\right)}^{2}\right)$	[0,1]	dim = 6 popsize = 100 iteration = 200
F5	$F\left(x\right)=-\sum_{i=1}^{4}c_{i}\exp\left(-\sum_{j=1}^{3}a_{ij}{\left(x_{j}-p_{ij}\right)}^{2}\right)$	[1,3]	dim = 3 popsize = 100 iteration = 120
F6	$F\left(x\right)={\left(\frac{1}{500}+\sum_{j=1}^{25}\frac{1}{j+\sum_{i=1}^{2}{\left(x_{i}-a_{ij}\right)}^{6}}\right)}^{-1}$	[−65,65]	dim = 2 popsize = 100 iteration = 180

**Table 4:** Simulation results of algorithms.

	GWO	PSO	WOA	SSA	IBES	Theoretical Value
F1	−10.5364	−105364	−10.5364	−10.5364	**−10.5364**	**−10**
F2	−10.4042	−10.4029	−10.4029	−10.4029	**−10.4029**	**−10**
F3	−10.1561	−10.1532	−10.1576	−10.1532	**−10.1532**	**−10**
F4	−3.3220	−3.3311	−3.3231	−3.3220	**−3.3220**	**−3**
F5	−3.8628	−3.8628	−3.8627	−3.8628	**−3.8628**	**−3**
F6	0.9980	0.9980	0.9980	2.9821	**0.9980**	**1**

**Table 5:** Data after processing.

Number of Coefficient	Auxiliary Variable	Correlation
1	Influent BOD	0.9179
2	Effluent COD	0.9151
3	Influent TN	0.9119
4	Effluent pH	0.8878
5	Influent NH3-N	0.8826
6	Influent pH	0.8716
7	Influent COD	0.8676
8	Influent Chroma	0.8669
9	Influent oil	0.8562
10	Effluent SS	0.8556
11	Effluent oil	0.8519
12	Effluent Chroma	0.8415
13	Influent phosphate	0.8397
14	MLSS in biochemical tank	0.8037

**Table 6:** Predictive index of BOD.

Model	MSE	RMSE	MAE	R${}^{2}$
CNN	0.0847	0.1500	0.1115	0.9503
LSTM	0.1310	0.2985	0.2330	0.8132
ELMAN	0.2425	0.3120	0.2523	0.7849
GWO-LSSVM	0.0659	0.0217	0.0182	0.9889
WOA-LSSVM	0.0711	0.1831	0.1521	0.9693
PSO-LSSVM	0.0587	0.1049	0.0851	0.9757
SSA-LSSVM	0.0726	0.2371	0.1707	0.9758
**IBES-LSSVM**	**0.0201**	**0.0104**	**0.0103**	**0.9911**

**Table 7:** PI of BOD.

	μ = 90%					μ = 95%					μ = 99%				
	PICP	PINRW	CWC	PINAW	Time	PICP	PINRW	CWC	PINAW	Time	PICP	PINRW	CWC	PINAW	Time
CNN	0.9298	0.2731	0.2731	0.2348	41.489	0.9617	0.3848	0.3848	0.3325	42.940	0.9911	0.2841	0.2841	0.2413	46.076
LSTM	0.9124	0.3632	0.3632	0.3112	27.486	0.9609	0.3796	0.3796	0.3254	27.731	0.9913	0.3554	0.3554	0.3020	27.821
ELMAN	0.9073	0.2978	0.2978	0.2474	316.316	0.9549	0.2573	0.2573	0.2202	241.446	0.9909	0.2571	0.2571	0.2132	90.582
WOA-LSSVM	0.9104	0.2663	0.2663	0.2325	1.686	0.9633	0.2697	0.2697	0.2346	1.873	0.9909	0.2673	0.2673	0.2245	1.654
GWO-LSSVM	0.9099	0.2557	0.2557	0.2241	1.396	0.9587	0.2668	0.2668	0.2355	1.389	0.9911	0.2689	0.2689	0.2254	2.012
PSO-LSSVM	0.9111	0.2519	0.2519	0.2198	1.029	0.9544	0.2596	0.2596	0.2155	0.967	0.9908	0.2773	0.2773	0.2277	0.963
SSA-LSSVM	0.9072	0.2901	0.2901	0.2543	1.428	0.9563	0.3178	0.3178	0.2613	1.410	0.9907	0.2961	0.2691	0.2245	1.599
**IBES-LSSVM**	**0.9053**	**0.2468**	**0.2468**	**0.2007**	**1.406**	**0.9531**	**0.2569**	**0.2569**	**0.2064**	**1.432**	**0.9907**	**0.2569**	**0.2569**	**0.2111**	**1.207**

**Table 8:** Data after processing.

Number of Coefficient	Auxiliary Variable	Correlation
1	Influent TP	0.8730
2	Anaerobic terminal ORP	0.8726
3	Effluent PH	0.8693
4	Temperature	0.8659
5	Total suspended solids TTS	0.8525
6	Effluent ORP	0.8257
7	Effluent nitrate	0.8143

**Table 9:** PI of NH3-N.

	μ = 90%					μ = 95%					μ = 99%				
	PICP	PINRW	CWC	PINAW	Time	PICP	PINRW	CWC	PINAW	Time	PICP	PINRW	CWC	PINAW	Time
CNN	0.9231	0.53951	0.53951	0.50111	29.991	0.9619	0.49776	0.49776	0.46854	32.446	0.9919	0.52063	0.52063	0.48445	31.703
LSTM	0.9182	0.49437	0.49437	0.44235	22.176	0.9588	0.42320	0.42320	0.37824	22.637	0.9921	0.53185	0.53185	0.50111	21.272
ELMAN	0.9066	0.38637	0.38637	0.34255	6.661	0.9580	0.37625	0.37625	0.32142	3.175	0.9912	0.42032	0.42032	0.38764	3.120
WOA-LSSVM	0.9197	0.49711	0.49711	0.45739	1.547	0.9581	0.46106	0.46106	0.42131	1.711	0.9913	0.47562	0.47562	0.41121	1.584
GWO-LSSVM	0.9227	0.51067	0.51067	0.46174	1.346	0.9601	0.51117	0.51117	0.47894	1.166	0.9913	0.51776	0.51776	0.45669	1.163
PSO-LSSVM	0.9241	0.48209	0.48209	0.45394	0.959	0.9604	0.47815	0.47815	0.42756	0.797	0.9917	0.49209	0.49209	0.46401	0.801
SSA-LSSVM	0.9112	0.40579	0.40579	0.35752	1.363	0.9574	0.38947	0.38947	0.34556	1.184	0.9909	0.38777	0.38777	0.36454	1.142
**IBES-LSSVM**	**0.9037**	**0.34531**	**0.34531**	**0.30989**	**1.354**	**0.9556**	**0.34906**	**0.34906**	**0.31128**	**1.181**	**0.9907**	**0.34677**	**0.34677**	**0.31001**	**1.366**

**Table 10:** Predictive index of NH3-N.

Model	MSE	RMSE	MAE	R${}^{2}$
CNN	0.1874	0.1711	0.1450	0.8932
LSTM	0.1138	0.2131	0.1663	0.7666
ELMAN	0.0954	0.1846	0.1564	0.7872
GWO-LSSVM	0.0997	0.0895	0.0628	0.7280
WOA-LSSVM	0.1929	0.2371	0.1709	0.8959
PSO-LSSVM	0.1312	0.1722	0.1247	0.8922
SSA-LSSVM	0.1196	0.1958	0.2037	0.8117
**IBES-LSSVM**	**0.0917**	**0.0645**	**0.0450**	**0.8967**

## Data Availability

Data are available upon request.
